# Programmable self-destruction of artificial cells with death signaling

**DOI:** 10.1039/d5sc08882h

**Published:** 2025-12-08

**Authors:** Joshua Krehan, Lorena Baranda Pellejero, Andreas Walther

**Affiliations:** a Life-Like Materials and Systems, Department of Chemistry, University of Mainz Duesbergweg 10-14 55128 Mainz Germany Andreas.Walther@uni-mainz.de

## Abstract

Programmed cell death is a crucial biological process that removes damaged or no longer needed cells and is orchestrated through complex intracellular signaling cascades. Mimicking such behavior in synthetic systems enables programmed disassembly after completing a task or releasing cargo on demand. Despite advances in engineering artificial cells (ACs) that mimic key cellular functions such as metabolism, homeostasis or communication, systems with a programmable lifetime remain unrealized. Here, we introduce a time-programmed self-destruction mechanism in ACs based on pH-responsive giant unilamellar vesicles, equipped with an internal UV-inducible acidification cascade. Upon light activation, photocaged glucose is enzymatically converted to gluconic acid, lowering the internal pH and destabilizing the pH-sensitive membrane, ultimately causing complete membrane collapse. The self-destruction is spatially confined and tunable in time, ranging from minutes to over an hour, depending on the light intensity. Furthermore, we demonstrate that collapse-induced release of DNA signals triggers defined downstream responses in neighboring ACs, including membrane labeling and aggregation. Our findings pave the way for ACs with programmed lifetimes, capable of on-demand release or disassembly in response to defined stimuli, allowing transmission of signals within complex synthetic environments.

## Abstract 2

We report artificial cells with a tunable, light-activated kill switch that drives irreversible collapse and initiates DNA-based signaling to neighboring receiver cells.

## Graphical abstract

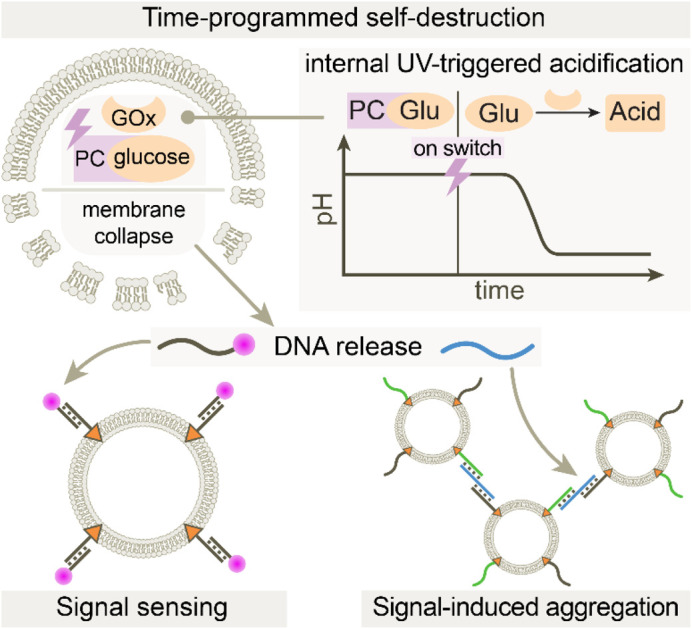

## Introduction

Programmed cell death is a tightly regulated biological process through which multicellular organisms remove damaged or unnecessary cells in response to internal or external signals.^1–3^ These signals trigger intracellular biochemical cascade reactions that lead to the controlled disassembly of cells and enable the release of molecular messengers, which then often activate downstream immune responses or regenerative programs in neighboring cells.^4–6^ Mimicking such behavior in synthetic systems holds potential for engineering life-like materials with autonomous disassembly, controlled lifetimes, or programmable communication. Artificial cells (ACs) have been engineered to replicate key biological functions such as controlled growth and division,^7–10^ response to their environment,^11–14^ and basic intercellular communication.^15–18^ More complex communication has further enabled programmable signal transport^19^ and DNA-based catalytic networks for chemical adaptation,^20^ and has recently been extended to AC-based spheroids in which inter-AC communication and homeostasis emerge as collective properties.^21^ While external light-induced bursting of lipid vesicles has been demonstrated using photosensitive surfactants,^22^ ACs that undergo a programmed, cell death-like disassembly *via* an internal mechanism, resulting in complete collapse, have not yet been achieved.

Enzymatic pH-feedback mechanisms enable autonomous pH control by coupling substrate consumption with the pH dependency of enzyme activity.^23^ Two prominent examples of pH-modulating enzymes are urease, hydrolyzing urea to ammonia and carbon dioxide and thereby increasing the pH, and glucose oxidase (GOx), converting glucose into gluconic acid and thereby lowering the pH.^24–26^ Beyond operation in plain solution, such enzymes have been compartmentalized in hydrogels,^27,28^ polymer beads,^29^ polymer capsules,^21^ liposomes,^30–32^ and polymersomes,^33–38^ where they effectively adjust their surrounding pH while maintaining stability of the compartments. To date, no mechanism has been established that triggers pH modulation strictly from within a compartment, which is related to the challenge of keeping a system dormant during assembly and enabling external triggering at defined times. To this end, photochemical uncaging of substrates for enzymes can provide an important spatiotemporal control mechanism.^39–41^

Herein, we introduce a time-programmed AC kill switch that drives the controlled self-destruction of ACs based on internal, UV-inducible pH modulation coupled to a pH-responsive giant unilamellar vesicle-based (GUV) chassis. Importantly, our design ensures that ACs remain stable until the self-destruction mechanism is activated by UV irradiation, after which they undergo irreversible collapse. The timing of collapse can be programmed from minutes to hours by tuning the UV intensity, and the response is spatially confined such that only ACs within the irradiated area self-destruct. We further demonstrate downstream signaling by showing that DNA released after self-destruction can be sensed on the membrane of intact receiver ACs and can even drive aggregation of ACs.

## Results

**Fig. 1:**
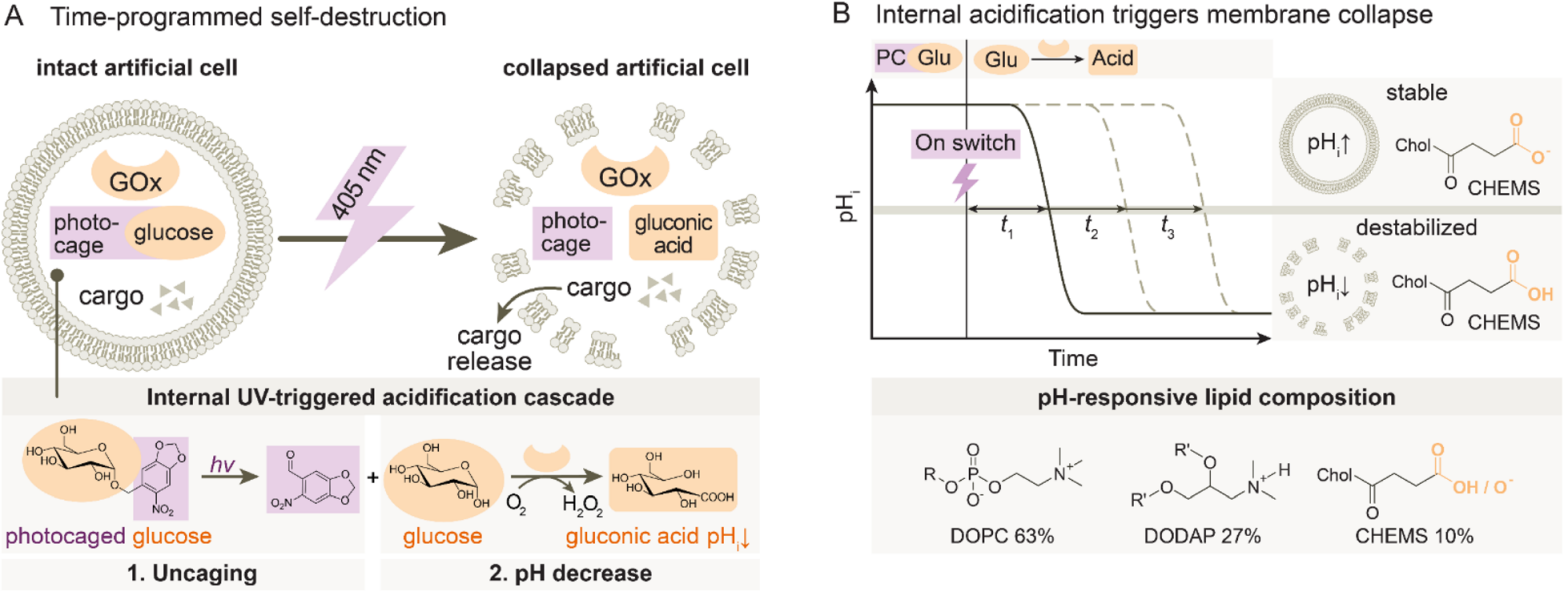
Time-programmed self-destruction of ACs. (A) Upon 405 nm UV irradiation, photocaged glucose is uncaged and enzymatically oxidized by GOx; glucose and O_2_ are converted into gluconic acid and H_2_O_2_, leading to a pH drop inside the AC. The internal acidification destabilizes the membrane, causing membrane collapse and cargo release. (B) The drop in pH destabilizes the pH-responsive membrane by protonating CHEMS due to disruption of the charge balance.

Our system design relies on pH-responsive GUV-based ACs encapsulating a UV-inducible acidification cascade by co-loading a photocaged glucose derivative (PCGlu)^42,43^ together with GOx (Fig. 1A). Upon UV irradiation, glucose is released from its photocaged form and subsequently converted by GOx to generate glucono-1,5-lactone, which rapidly hydrolyzes into gluconic acid, resulting in a gradual internal acidification. The GUV membrane is engineered with a lipid mixture consisting of DOPC (neutral fluid lipid), DODAP (cationic lipid), and CHEMS (cholesteryl hemisuccinate, anionic pH-sensitive lipid) (Fig. 1B).^44^ At neutral pH, the membrane is stable due to the electrostatic balance between the lipids. However, once the internal pH (pH_i_) drops below a threshold at slightly acidic pH, protonation of CHEMS disrupts the charge balance of the membrane, triggering destabilization and collapse with complete release of the encapsulated content.

**Fig. 2:**
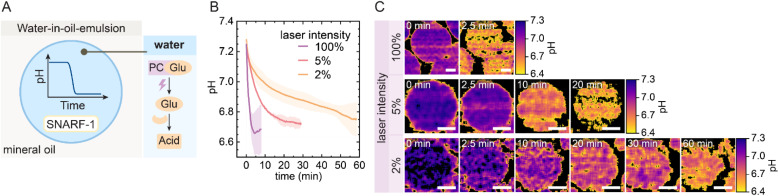
Controlled acidification in water-in-oil emulsions *via* laser-triggered glucose generation. (A) Schematic of the emulsion setup. (B) Kinetics of pH decrease at different 405 nm laser intensities (100%, 5%, 2%). Droplets contain 10 mM PCGlu, 1 g per L GOx, and 3 µM of the fluorescent pH indicator SNARF-1-dextran in PBS buffer (10 mM, pH 7.3). Shaded areas indicate standard deviation from two independent experiments. (C) Color-mapped CLSM images of pH changes in individual droplets over time at varying laser intensities. Black regions inside droplets indicate pixels for which the calculated pH values fell outside the reliable calibration range (pH 6.4–7.3). Scale bars: 25 µm.

We first demonstrate the UV-triggered, temporally programmable acidification of the PCGlu-GOx system in a simplified model using water-in-oil emulsions under conditions that mimic the internal environment of ACs (Fig. 2A). To monitor pH changes upon UV activation, we used 10 mM PCGlu, 1 g per L GOx, and 3 µM SNARF-1-dextran in PBS-buffered droplets (phosphate-buffered saline, 10 mM, pH 7.3). SNARF-1 is a ratiometric fluorescent pH indicator compatible with confocal laser scanning microscopy (CLSM). This enables imaging of individual water droplets or ACs and simultaneous pH measurement. During the experiment, the 405 nm laser of the CLSM continuously uncages PCGlu to release glucose. The kinetics of acidification depend directly on laser intensity: at 100%, the pH drops from ∼7.2 to ∼6.7 within 2 min, while 5% and 2% intensities result in the same final pH after ∼30 min and ∼60 min, respectively (Fig. 2B). Fluorescence-based pH maps of individual droplets confirm these trends and visualize the temporal progression of acidification (Fig. 2C). Together, these results demonstrate that the UV-triggered PCGlu-GOx system enables a tunable pH drop in confined compartments, with acidification timescales adjustable from minutes to over an hour depending on laser intensity.

**Fig. 3:**
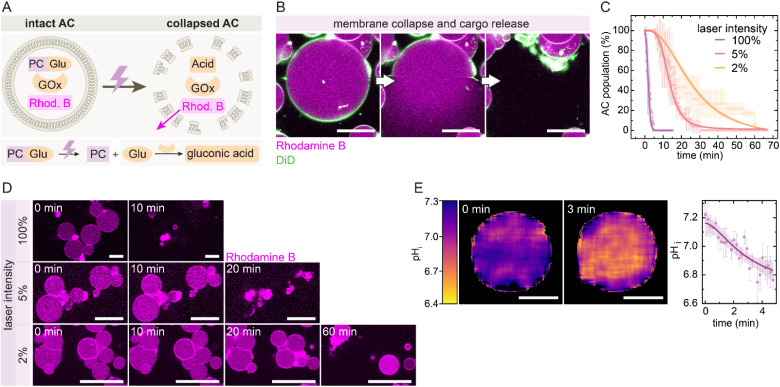
Time-programmed AC self-destruction *via* UV-triggered internal acidification and membrane collapse. (A) Schematic of the AC self-destruction mechanism. (B) CLSM images showing membrane destabilization and cargo release from ACs labeled with DiD (green, membrane) and Rhodamine B (magenta, core). Collapse happens within milliseconds. Scale bars: 25 µm. (C) Population decrease of ACs over time at different 405 nm laser intensities (100%, 5%, 2%). ACs contain 10 mM PCGlu, 1 g per L GOx, and 30 µM Rhodamine B in PBS buffer (10 mM, pH 7.3). Data points represent mean values from three independent experiments with ≥20 ACs in total, shown with standard deviation as error bars. Curves represent sigmoidal fits with 95% confidence bands indicated as shaded areas. (D) Time-lapse CLSM images showing AC population decrease at different laser intensities. Scale bars: 50 µm. (E) (Left) pH mapping of individual ACs using 3 µM SNARF-1-dextran in PBS buffer (10 mM, pH 7.3) internally shows rapid internal acidification. Scale bars: 25 µm. (Right) Quantification of pH drop. Data points represent mean values from three ACs, with standard deviation as error bars. Curves represent sigmoidal fits with 95% confidence bands indicated as shaded areas.

We then applied this system in GUV-based ACs, prepared by the phase transfer method,^45,46^ to demonstrate UV-triggered self-destruction and cargo release (Fig. 3A). ACs were loaded with 10 mM PCGlu, 1 g per L GOx, and 30 µM of the fluorescent cargo Rhodamine B, which enables visualization of both membrane integrity and release behavior. Upon UV activation, the internal reaction lowers the pH_i_, resulting in leakage of cargo and complete membrane collapse (Fig. 3B). Time-lapse CLSM confirms that the fraction of intact ACs decreases over time as a function of the laser intensity (Fig. 3C and D, SI Movie 1). At 100% laser intensity, most ACs collapse within 5 min, while 5% and 2% intensities lead to delayed collapse over 30–60 min. The collapse correlates with a measurable drop in pH_i_, as calculated from the encapsulated SNARF-1 dye (Fig. 3E). Notably, the pH_i_ decreases from ∼7.2 to ∼6.8 within 3 min at 100% intensity and thus reaches the critical range for CHEMS protonation and membrane destabilization. Control experiments confirm that the collapse mechanism is driven by internal acidification and depends on the pH-responsive lipid composition, as well as on parameters of the internal acidification cascade: no self-destruction occurs without GOx (SI Fig. S1A) or with non-pH-responsive DOPC membranes (SI Fig. S1B). Importantly, the generated H_2_O_2_ does not induce critical membrane oxidation or collapse, as further evidenced by the stability of DOPC ACs containing PCGlu and GOx. Note that H_2_O_2_ is a poor oxidant for lipid oxidation in the absence of catalysts.^47,48^ Low enzyme loading (≤0.001 g per L GOx, SI Fig. S1C), limited substrate concentration (≤4 mM PCGlu, SI Fig. S1D), or low CHEMS content (≤5% CHEMS, SI Fig. S1E) prevent collapse, while excessive CHEMS results in low vesicle yield during fabrication and predominantly permeable ACs (≥15% CHEMS, SI Fig. S1F). Moreover, the UV-triggered collapse can be spatially confined: by irradiating a defined region, only ACs within that area collapse, while neighboring ACs remain intact (SI Fig. S2). This underscores that a supercritical local acidification triggers the collapse, whereas its further dilution into the surrounding medium prevents the destruction of neighboring ACs. Together, these results confirm that UV-triggered acidification provides a reliable and spatially addressable mechanism to trigger time-programmed AC self-destruction.

**Fig. 4:**
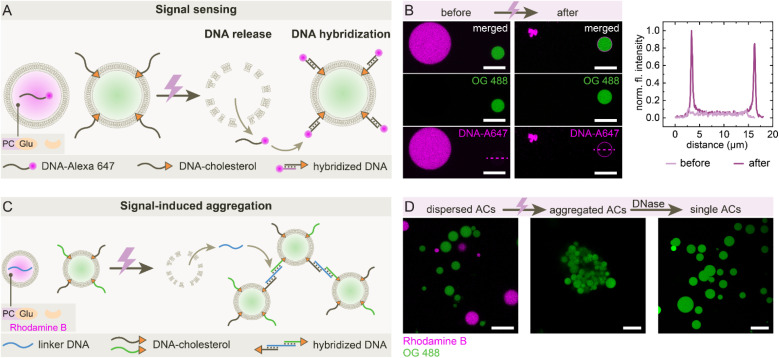
AC self-destruction induces DNA-mediated death signaling and aggregation. (A) Schematic of signaling: upon UV-triggered self-destruction of sender ACs, the internal DNA-A647is released and immediately captured by neighboring receiver ACs through hybridization with the complementary DNA-cholesterol strands anchored to their membranes. (B) CLSM images before and after UV irradiation showing DNA-A647 transfer to green-labeled OG 488 (Oregon Green 488) ACs. Self-destructing ACs contain 10 mM PCGlu, 1 g per L GOx, and 10 µM DNA-A647. Line profile of receiver AC confirms capture of released DNA-A647 at the membrane. Scale bars: 20 µm. (C) Schematic of death-signal-induced aggregation: DNA release triggers crosslinking *via* linker DNA and two membrane-anchored DNA-cholesterol strands, leading to aggregation of receiver ACs. (D) CLSM images showing individual receiver ACs and sender ACs before irradiation (left), self-destructed senders (not visible) and aggregated receiver ACs post UV (middle), and redispersed receiver ACs after DNase treatment (right). Self-destructing sender ACs contain 10 mM PCGlu, 1 g per L GOx and 10 µM linker DNA. At least 10 linker DNA-containing ACs were irradiated and after self-destruction, the ACs were gently mixed with a pipette to initiate aggregation. Scale bars: 20 µm.

Finally, we designed a system in which UV-triggered self-destruction of sender ACs leads to signal release and downstream responses in neighboring receiver ACs, which do not feature the self-destruction mechanism (Fig. 4A). Sender ACs encapsulate the PCGlu-GOx system and a single-stranded DNA labeled with Alexa 647 (DNA-A647), while receiver ACs are functionalized with membrane-anchored complementary DNA-cholesterol. Upon UV activation, the sender ACs self-destruct and release DNA-A647 into the local environment. The released DNA-A647 then hybridizes with its complementary strand on the receiver AC membrane, resulting in localized membrane fluorescence (Fig. 4B). Line profile analysis confirms this transition. Building on this signaling, we implemented a second response module involving a linker DNA to mediate signal-induced aggregation (Fig. 4C). Upon UV activation, the released DNA bridges receiver ACs *via* hybridization with DNA-cholesterol on their surfaces, leading to visible AC clustering (Fig. 4D, further overview images of clustering in SI Fig. S3). The aggregation can be reversed with DNase treatment, confirming the DNA-based mechanism. Together, these findings demonstrate that ACs can be equipped with communication modules based on self-destruction, capable of releasing molecular signals to elicit specific death responses in their environment.

## Conclusion

In summary, we have introduced a temporally programmable cell death-like AC self-destruction mechanism based on an internally triggered, light-inducible acidification cascade that enables the irreversible collapse of specifically engineered GUV-based ACs. By combining photocaged glucose and glucose oxidase with a pH-sensitive lipid membrane, we achieved a controlled and tunable drop in internal pH, resulting in membrane destabilization and complete AC disassembly. The collapse process is irreversible, spatially confined, and can be precisely timed from minutes to over an hour by adjusting the light intensity. Beyond that, we demonstrated that AC self-destruction can be coupled to downstream signaling, including membrane-localized signal sensing and aggregation in neighboring intact ACs, thereby establishing a synthetic communication pathway initiated by self-destruction. Looking forward, this concept lays the foundation for the development of self-regulating artificial tissues, signal-propagating prototissues, and responsive material systems with controllable lifetimes and programmed disassembly.

## Author contributions

Conceptualization, J. K., and A. W.; methodology, J. K. and L. B. P.; writing – original draft, J. K.; writing – review and editing, J. K., L. B. P., and A. W.; funding acquisition, A. W.; resources, A. W.; supervision, A. W.

## Conflicts of interest

The authors declare no competing interests.

## Supplementary Material

SC-017-D5SC08882H-s001

SC-017-D5SC08882H-s002

## Data Availability

The datasets in the current study are available from the corresponding author on reasonable request. Supplementary information (SI): all experimental details, procedures, and supporting data for this article. See DOI: https://doi.org/10.1039/d5sc08882h.
