# Supplementary material for: Programmable self-destruction of artificial cells with death signaling
Source: Chem Sci. 2025 Dec 8;17(4):2372–7. doi: 10.1039/d5sc08882h (PMC12683541; doi:10.1039/d5sc08882h)
Supplement: SC-017-D5SC08882H-s002 [file SC-017-D5SC08882H-s002.pdf]

Supplementary Information (SI) for Chemical Science.
This journal is © The Royal Society of Chemistry 2025

Supplementary Information

Programmable Self-Destruction of Artificial Cells with Death Signaling

Joshua Krehan,[1] Lorena Baranda Pellejero,[1] and Andreas Walther[1,2,]*

[1]Life-Like Materials and Systems, Department of Chemistry, University of Mainz, Duesbergweg 10-14, 55128 Mainz, Germany

[2]Lead contact

*Correspondence: Andreas.Walther@uni-mainz.de

Contents

S1 Materials and Methods 3

Materials. 3

Image acquisition. 3

Synthesis of photocaged glucose (PCGlu, 6-nitropiperonyl α/β-D-glucopyranoside). 3

UV-induced pH decrease in water-in-oil emulsions. 3

Preparation of liposomal ACs and self-destructing ACs. 4

pH calculation and color mapping in ACs and water-in-oil emulsion. 4

Signal sensing and signal-induced aggregation. 4

S2 Supplementary Tables 5

S3 Supplementary Figures 5

S4 References 6

S1 Materials and Methods

Materials. Mineral Oil (BioUltra for molecular biology, 69794, Sigma-Aldrich), ABIL® EM 180 (Evonik Industries AG), dextran SNARF™-1 (SNARF-1-dextran, anionic, M_w 70,000, InvitrogenTM, D3304, Thermo Fisher Scientific), Dulbecco's Phosphate Buffered Saline (DPBS, modified, without calcium chloride and magnesium chloride, D8537, Sigma-Aldrich), glucose oxidase (GOx, from aspergillus niger, Type X-S, 100,000–250,000 units/g solid without added oxygen, G7141, Sigma-Aldrich), nitropiperonyl alcohol (98%, A18140.14, Thermo Fisher Scientific), acetobromo-α-D-glucose (95%, A1750, Sigma-Aldrich), silver carbonate (Ag_2CO_3, 99%, 179647, Sigma-Aldrich), silver trifluoromethanesulfonate (AgOTf, 99.95%, 483346, Sigma-Aldrich), dichloromethane (extra dry, 99.9%, 326850025, Thermo Fisher Scientific), molecular sieves (3 Å, 0.3 mm, Type 564, 8487.1, Carl Roth), ammonia (7 N solution in methanol, 499145, Sigma-Aldrich), poly(vinyl alcohol) (M_w 85,000–124,000, 99+% hydrolyzed, 363146, Sigma-Aldrich), 1,2-di-(9Z-octadecenoyl)-sn-glycero-3-phosphocholine (DOPC, 25 mg/mL solution in chloroform, 850375C, Sigma-Aldrich), 3β-Hydroxy-5-cholestene 3-hemisuccinate; 5-Cholesten-3β-ol 3-hemisuccinate (CHEMS, 99%, 850524P, Sigma-Aldrich), 1,2-dioleoyl-3-dimethylammonium-propane (DODAP, 25 mg/mL solution in chloroform, 890850C, Sigma-Aldrich), 1,1'-dioctadecyl-3,3,3',3'-tetramethylindodicarbocyanine 4-chlorobenzenesulfonate salt (DiD, D7757, Thermo Fisher Scientific), Oregon Green™ 488 Cadaverine, 5-isomer (OG488, O10465, Thermo Fisher Scientific), rhodamine B (83689, Sigma-Aldrich), Optiprep™ (60 w/v% iodixanol in water, 17109821, Thermo Fisher Scientific), DNase (Deoxyribonuclease I, M0303S, New England Biolabs).

Image acquisition. Confocal laser scanning microscopy (CLSM) was performed using a Leica Stellaris 5 microscope equipped with LasX software (v4.3.0.24308). Imaging was performed with four laser lines and three HyD S detectors, employing a 63× oil immersion objective (PL APO, 1.40 NA) and a 5× dry objective (PL FLUOTAR, 0.15 NA). Images were acquired at 2048 × 2048 pixels and a scan speed of 600 Hz. GUV were imaged in glass-bottom 96 well plates, which were coated with poly(vinyl alcohol) (PVA, M_w 85,000–124,000, 99+% hydrolyzed) by adding 20 µL of a 2.5% PVA solution in water, removing the solution and heating the well plate for 20 min at 70 °C.

Synthesis of photocaged glucose (PCGlu, 6-nitropiperonyl α/β-D-glucopyranoside). Two Schlenk tubes were each charged with 2.40 g of activated 3 Å molecular sieves. In the first tube, 1.00 g of acetobromo-α-D-glucose (2.3 mmol, 1.0 eq) was dissolved in 20 mL of dry dichloromethane, while in the second tube, 1.20 g of nitropiperonyl alcohol (6.1 mmol, 2.5 eq) was dissolved in 20 mL of dry dichloromethane. Both solutions were stirred at room temperature for 30 min before being combined. Subsequently, 0.40 g of Ag_2CO_3 (1.5 mmol, 0.6 eq) and 0.37 g of AgOTf (1.5 mmol, 0.6 eq) were added, and the reaction mixture was stirred overnight at room temperature under nitrogen and in the dark. The resulting precipitates and molecular sieves were removed by filtration, the filtrate was washed with saturated $NaHCO_3$ solution, dried over $MgSO_4$, and the solvent was removed under reduced pressure. The crude residue was dissolved in 50 mL of 7 N ammonia in methanol and stirred at room temperature overnight. After evaporation of the solvent, the product was purified by column chromatography using a 1:1 mixture of ethyl acetate and cyclohexane.

UV-induced pH decrease in water-in-oil emulsions. The aqueous phase consisted of DPBS supplemented with 10 mM PCGlu, 1 g/L GOx and 3 µM SNARF-1-dextran. The oil phase was prepared by dissolving 2 wt% ABIL® EM 180 in mineral oil. To generate water-in-oil emulsions, 20 µL of the aqueous phase was mixed with 450 µL of the oil phase and vortexed for 30 s. Emulsions were using CLSM, and uncaging of the PCGlu was performed directly via the 405 nm laser of the microscope. Laser intensities of 100%, 5% and 2% were applied continuously during acquisition.

Preparation of liposomal ACs and self-destructing ACs. Liposomal ACs were prepared using a phase-transfer method.[1] A lipid stock mixture consisting of DOPC:DODAP:CHEMS:DiD (63:27:10:0.1 mol%, if not mentioned otherwise) at a total concentration of 25 mg/mL was prepared. The inner solution consisted of 18 w/v% iodixanol (0.3× Optiprep™) in PBS (10 mM, pH 7.3), including: 10 mM PCGlu, 1 g/L GOx, 30 µM rhodamine B (Figure 3B, 3C, 3D; SI Figure S1B, S1E, S1F, S2); 10 mM PCGlu, 0.001 g/L GOx, 30 µM rhodamine B (SI Figure S1C); 10 mM PCGlu, 1 g/L GOx, 3 µM SNARF-1-dextran (Figure 3E); 10 mM PCGlu, 1 g/L GOx, 10 µM DNA-A647 (Figure 4B); 10 mM PCGlu, 1 g/L GOx, 10 µM linker DNA (Figure 4D); 10 µM OG488 (Figure 4B, 4D); 10 mM PCGlu, 30 µM rhodamine B (SI Figure S1A); 4 mM PCGlu, 1 g/L GOx, 30 µM rhodamine B (SI Figure S1D). In a 5 mL glass vial, 70 µL of the lipid stock solution was added and dried under a nitrogen stream to form a thin lipid film. Subsequently, 700 µL of dry mineral oil was added, the mixture was vortexed for 15 s, and then sonicated at 55 °C for 20 min to fully dissolve the lipids. After the solution reached room temperature, 7 µL of the inner solution was added to the lipid-in-oil mixture and vortexed thoroughly (4000 rpm, 1 min) to generate a stable water-in-oil emulsion. The emulsion was immediately poured onto 500 µL DPBS outer solution in a 2 mL Eppendorf tube. The tube was then centrifuged at 4500 × g for 10 min at 4 °C. After centrifugation, the upper oil phase and debris were carefully removed. The remaining ~200 µL aqueous phase containing the pellet was gently resuspended by pipetting up and down 5–8 times with a 100 µL pipette. Liposomal ACs were imaged in 96-well glass-bottom plates that had been pre-coated with PVA. To this end, 80 µL of a 2 wt% PVA solution in water were added to each well, the solution was removed right after leaving a thin layered coating in the glass surface, and the plates were heated at 70 °C for 15 min to dry the remaining PVA film. For imaging, 60 µL of sample was added per well. In AC self-destruction experiments shown in Figure 3, 15 µL of a 2 wt% agarose solution in water (final concentration 0.4 wt%) was added to the sample to reduce AC movement and facilitate imaging. The internal acidification cascade was triggered using the 405 nm laser of the CLSM. Laser intensities of 100%, 5%, and 2% were applied continuously during image acquisition.

pH calculation and color mapping in ACs and water-in-oil emulsion. Images were recorded using an excitation wavelength of 552 nm, with emission channels at 565–595 nm (em_1) and 625–655 nm (em_2), corresponding to the two pH-dependent emission maxima of SNARF-1. For calibration, single images of buffer solutions ranging from pH 5.5 to 7.6 were acquired under identical settings. Analysis was performed using a custom Python script. For each image, the script calculated the intensity ratio (em_1/em_2) of each pixel, excluding black pixels. A mean ratio was determined per image, and a sigmoidal calibration curve was generated from the buffer series. This curve was used to convert the emission ratio to pH values for each frame in time-lapse recordings. For color-mapped pH visualization, a Python script was used that calculated the pH values from the emission ratio as described before and visualized each pixel as a corresponding color using an inverted plasma colormap. A 30 × 30-pixel sliding window filter was applied to locally average non-black pixels, and pixels with zero intensity were replaced by the global mean of the non-zero pixel values.

Signal sensing. Two types of ACs were prepared: a self-destructing type containing 10 mM PCGlu, 1 g/L GOx, and 10 µM DNA-A647, and a second type containing only 10 µM OG488 dye. For the experiment, 60 µL of the OG488-containing ACs were added to a well together with 100 nM DNA-cholesterol and incubated for 5 min. Subsequently, 60 µL of the self-destructing ACs were added to the same well. The sample was imaged immediately using CLSM, and self-destruction was induced using 100% laser intensity of the 405 nm channel.

Signal-induced aggregation. Two types of ACs were prepared: a self-destructing type containing 10 mM PCGlu, 1 g/L GOx, and 10 µM linker DNA, and a second type containing only 10 µM OG488 dye. For the experiment, 60 µL of the OG488-containing ACs were added to a well together with 100 nM DNA-cholesterol and 100 nM DNA-cholesterol-B and incubated for 5 min. Subsequently, 60 µL of the self-destructing ACs were added to the same well and thoroughly

pipetted up and down once. The sample was imaged immediately using CLSM, and self-destruction of at least 10 ACs was induced using 100% laser intensity of the 405 nm channel. Afterwards, the sample was pipetted up and down once and imaged again.

S2 Supplementary Tables

Table S1. DNA sequences for the oligonucleotides purchased from IDT and Biomers with abbreviations and modifications. All oligonucleotides were purified via HPLC by the manufacturer.

Name	Sequence 5'→3'	Modification
DNA-A647	TAC TGA ACT CGA TAA TCA **AGT CTC ATA ATG GTT**	5' Alexa 647
DNA-cholesterol	**TGC GAG CCG TCC AA**C CAT TAT GAG ACT	3' Cholesterol-TEG
DNA-cholesterol-B	TCC GTC GTG CCT T**AT TTC TGA TGT CCA**	5' Cholesterol-Prolinol
linker DNA	**TT GGA CGG CTC GCA TGG ACA TCA GAA AT**	none

S3 Supplementary Figures

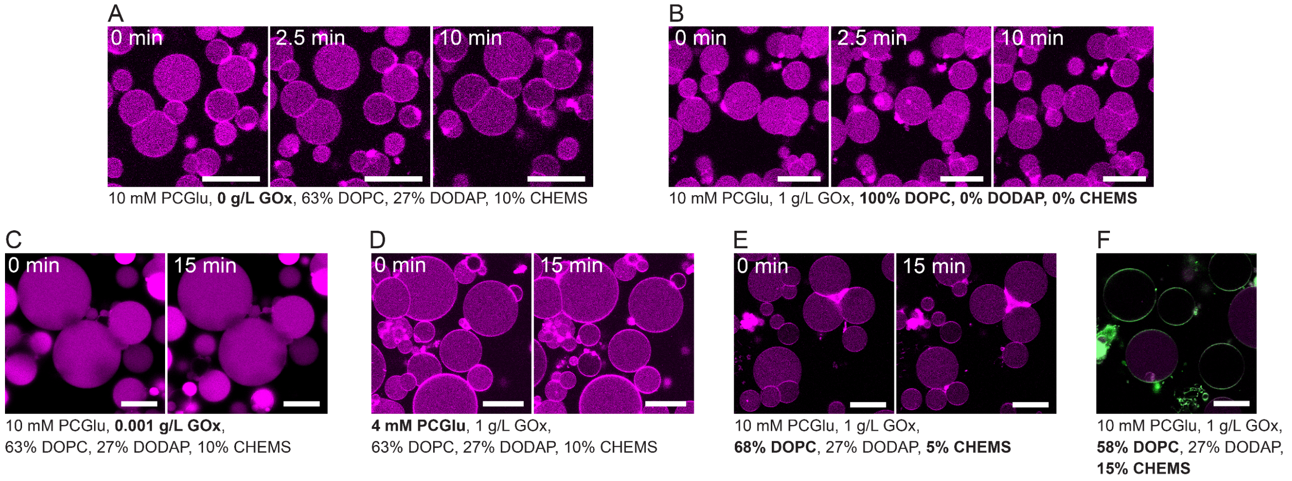

Figure S1. Control experiments confirm that collapse requires internal acidification and pH-responsive lipids. **A** No self-destruction occurs without GOx. **B** Non-pH-responsive DOPC membranes remain stable despite acid generation, also confirming that osmotic imbalance or H_2O_2 generation is not responsible for the collapse. **C** Low GOx concentration (0.001 g/L) is insufficient to trigger collapse. **D** Reduced PCGlu concentration (4 mM) prevents self-destruction. **E** Lower CHEMS content (5%) prevents collapse. **F** Higher CHEMS content (15%) reduces vesicle yield and forms permeable membranes that either lack internal rhodamine B (encapsulated as cargo, magenta, 30 µM) or exhibit only weak fluorescence. Membranes are stained with 0.1% DiD (green fluorescence). **A-F** ACs were continuously irradiated at 100% laser intensity at 405 nm. Magenta represents 30 µM rhodamine B in the interior. Scale bars: 10 µm.

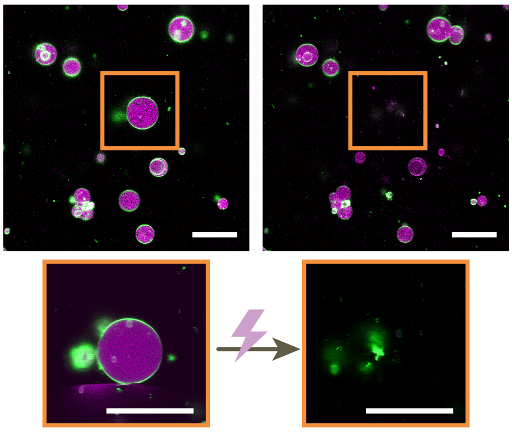

Figure S2. Localized irradiation demonstrates spatial control of time-programmed AC death. Only the area within the orange square was exposed to 405 nm light, resulting in selective collapse of ACs in the irradiated region. This confirms that the system enables spatially confined, time-programmed self-destruction. ACs contained 10 mM PC Glu, 1 g/L GOx, and 30 µM rhodamine B and the area was irradiated for 5 min. Membranes are stained with 0.1% DiD (green fluorescence). Scale bars: 25 µm.

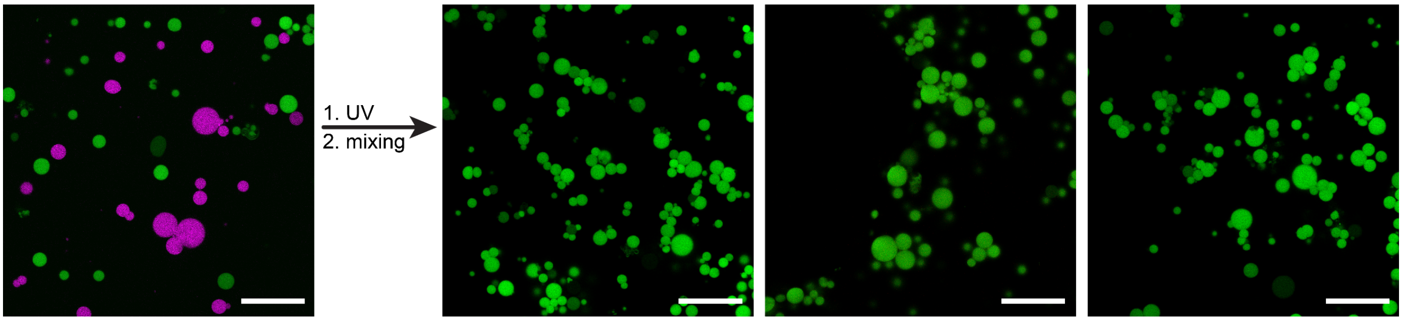

Figure S3. Additional CLSM images showing DNA-mediated aggregation of receiver ACs (green) after UV-triggered self-destruction of sender ACs (magenta). Following UV irradiation and destruction of magenta sender ACs, and gentle mixing, released linker DNA bridges neighboring receiver ACs functionalized with complementary DNA-cholesterol, leading to clustering. Images illustrate reproducibility of the aggregation behavior under identical experimental conditions (**Figure 4D**). Green corresponds to Oregon Green 488 in the receiver AC membranes, and magenta indicates rhodamine B in sender ACs before UV activation. Scale bars: 50 µm.

S4 References

1 Y. Shimane and Y. Kuruma, *Front. Bioeng. Biotechnol.*, DOI:10.3389/fbioe.2022.873854.
